# Fashion-Oriented Image Captioning with External Knowledge Retrieval and Fully Attentive Gates

**DOI:** 10.3390/s23031286

**Published:** 2023-01-23

**Authors:** Nicholas Moratelli, Manuele Barraco, Davide Morelli, Marcella Cornia, Lorenzo Baraldi, Rita Cucchiara

**Affiliations:** 1 Department of Engineering “Enzo Ferrari”, University of Modena and Reggio Emilia, 41125 Modena, Italy; 2 Department of Education and Humanities, University of Modena and Reggio Emilia, 42121 Reggio Emilia, Italy

**Keywords:** image captioning, fashion captioning, knowledge retrieval, vision-and-language

## Abstract

Research related to fashion and e-commerce domains is gaining attention in computer vision and multimedia communities. Following this trend, this article tackles the task of generating fine-grained and accurate natural language descriptions of fashion items, a recently-proposed and under-explored challenge that is still far from being solved. To overcome the limitations of previous approaches, a transformer-based captioning model was designed with the integration of external textual memory that could be accessed through *k*-nearest neighbor (*k*NN) searches. From an architectural point of view, the proposed transformer model can read and retrieve items from the external memory through cross-attention operations, and tune the flow of information coming from the external memory thanks to a novel fully attentive gate. Experimental analyses were carried out on the fashion captioning dataset (FACAD) for fashion image captioning, which contains more than 130k fine-grained descriptions, validating the effectiveness of the proposed approach and the proposed architectural strategies in comparison with carefully designed baselines and state-of-the-art approaches. The presented method constantly outperforms all compared approaches, demonstrating its effectiveness for fashion image captioning.

## 1. Introduction

Thanks to the growth of e-commerce websites and the increasing importance of fashion and style in our daily lives, the computer vision community has focused on fashion-related research over the past few years. As a consequence, multi-modal tasks, such as text-to-image generation [1] and cross-modal retrieval [2,3], have been extended and adapted to the fashion domain, resulting in virtual try-on approaches [4,5,6,7] and cross-modal retrieval methodologies of clothing items [8,9]. Image captioning, i.e., the task of generating natural language descriptions from visual inputs, streamlines and expedites the closed-captioning procedure for the creation and delivery of digital information and has, therefore, received significant interest from researchers [10]. On shopping websites, accurate and alluring descriptions of clothing could aid users in better understanding the features of clothing items and boost online sales by attracting more users.

Image captioning, despite its potential benefit, has not been widely studied in the fashion domain. This is due to the lack of publicly available datasets and the limitations of current captioning models, which in most cases are developed to work on natural images and perform poorly when applied to the fashion domain. The majority of captioning approaches [11,12,13], indeed, can only recognize high-level classes of objects and neglect the description of details. Moreover, generated captions are typically shorter than human-written ones, which again limits their applicability to fashion data, where longer descriptions are required. Lastly, they fail to generate words that do not appear (or have a low incidence) during the training phase and, therefore, struggle to manage specific and infrequent concepts.

A recent attempt to develop a fashion-oriented image captioning architecture was the model proposed by Yang et al. [14], which employs an LSTM language model trained with two reward functions, one related to the generation of single attributes and one that covers the semantics of the entire sentence. In this respect, the approach introduced in this manuscript takes a different path and aims at generating unbiased descriptions by retrieving additional information from an external source of textual data. Although the proposed approach can be directly employed for fashion item captioning, it can also be functional as a principal ingredient for other complex tasks, such as image generation from reference [15,16], and enable image–text retrieval of clothing items.

Specifically, a novel model for fashion-oriented image captioning was introduced, augmenting a sequence-to-sequence transformer with an external knowledge base accessible through a retrieval component. This provides an end-to-end model that can seamlessly read and integrate relevant information retrieved from the external memory and can improve the quality of the generated descriptions. This is achieved by pairing the classical multi-head attention mechanism on the progressively generated words with one that works on sentences and textual chunks that are extracted from the external memory. The contributions of these two attentive components are balanced through fully attentive gates.

To validate the effectiveness of the presented solution, several experiments were conducted on the fashion captioning dataset (FACAD) [14], which is the largest available dataset for fashion captioning. This, compared to datasets containing natural images, such as Microsoft COCO [17] and Conceptual Captions [18], features a more alluring expressive style that might pique customers’ curiosity, providing a high level of correlation with our task. Experimentally, the proposed approach is compared with carefully designed baselines, using different visual descriptors, validating the effectiveness of the introduced components and the usage of external memory.

To summarize, the key element proposed in this article is represented by the integration of external memory into self-attentive architecture, which provides direct access to relevant textual information and can ultimately improve the quality of generated descriptions. Experimentally, the effectiveness of the newly introduced retrieval stage is verified by varying the model in its architecture and visual features.

Overall, the rest of the paper is organized as follows: the next section will provide an overview of the related literature on image captioning and fashion-oriented solutions for vision and language; in Section 3, the proposed method for fashion image captioning will be introduced, focusing on the description of the knowledge retrieval architecture with fully attentive gates; finally, Section 4 will present experimental analyses of the key components of the proposed solution and comparisons with state-of-the-art approaches.

## 2. Related Work

In this section, a comprehensive review of the related literature on the image captioning task is reported, highlighting recent advances in the field of vision and language for the fashion domain.

### 2.1. Image Captioning

In the last decade, several research efforts have been made in the field of image captioning with a particular focus on the generation of textual descriptions for generic images [10]. In this context, initial approaches were based on the generation of simple template sentences, which were later filled by the output of an object detector or an attribute predictor [19,20]. With the advent of deep learning, captioning approaches have started to employ recurrent neural networks as language models using the output of a standard convolutional neural network (CNN) to encode input images and condition the generation of the output sentences [11,21,22].

After these initial attempts, subsequent methods have consistently improved both image encoding and language generation phases. On the image encoding side, notable achievements have been made with the integration of additive attention mechanisms to incorporate spatial knowledge, initially from a grid of CNN features [12], and then using image regions extracted with an object detector [13] eventually considering their semantic and spatial relationships encoded by graph neural networks [23]. Regarding the language generation phase, after the emergence of convolutional language models, which have also been explored for captioning [24], considerable advances have been achieved thanks to the introduction of transformer-like architectures [25,26]. While these models were initially proposed for machine translation and language understanding tasks, they were later applied to image captioning with the introduction of different captioning methods that incorporate fully attentive paradigms to improve the final performance [27,28,29,30]. Some of these works proposed self-attention only in the image encoding stage [27,28], while others introduced instead fully attentive architectures with self-attention mechanisms in both image encoder and language decoder modules [29,30,31,32,33,34]. Transformer-like architectures have also been applied directly on image patches, thus excluding or limiting the usage of the convolutional operator [35]. Some convolution-free attempts have been introduced for image captioning as well [36,37], using a pre-trained vision transformer network (i.e., ViT [35]) as an image encoder and employing a standard transformer decoder to generate textual descriptions. Other solutions [32,34] exploit self-attention to effectively combine visual features coming from multiple backbones (i.e., typically a CNN and an object detector), in some cases fine-tuning them to boost the final results [34].

In the last few years, image captioning models have achieved remarkable performance thanks to vision and language pre-training and early fusion strategies [38,39,40], as well as the introduction of large-scale multi-modal architectures, such as CLIP (“Contrastive Language-Image Pre-training”) [41] to obtain better image representations [33,42,43,44]. In this context, following the BERT (“Bidirectional Encoder Representations from Transformers”) architecture [26], a single stream of transformer layers was introduced in [38,39], where image regions, word tokens, and objects tags, extracted from an object detector, were fused into a unique flow. These architectures are usually pre-trained on large-scale data extracted from the web, even with the integration of specific techniques to explicitly ensure quality in web-scale training, such as learning image-grounded caption filters [40] or the separation of semantics and style by means of a special token [43]. To partially mitigate the need for large-scale training, a different approach is presented in [45] and other contemporary works [43,44] in which the captioning model is equipped with retrieval components that can enrich the semantics and quality of predicted textual sentences.

### 2.2. Fashion-Oriented Solutions for Vision and Language

In the last few years, many different solutions have been proposed for the fashion domain to make the e-commerce customer experience more effective and enjoyable including, for example, garment retrieval [46,47], clothing recommendations, and compatibility [48,49,50,51], and virtual try-on [4,5,6,7]. In this setting, the effective combination of multiple modalities (such as visual and textual data) has recently received a lot of attention with the introduction of several vision-and-language architectures specifically designed for the fashion domain [9,14,15,52,53]. Among them, some works focus on text-to-image retrieval and interactive fashion search [8,9], while others instead focus on image generation using textual sentences or attributes as input [15,16].

Multi-modal pre-training using large-scale fashion datasets is also effective in many downstream tasks. In this context, Zhuge et al. [52] proposed an alignment-guided masking strategy to jointly focus on image–text semantic relations and learn better vision and language embeddings. Differently, Mirchandani et al. [54] introduced a novel fashion-specific pre-training framework based on weakly supervised triplets, while in [53], two different pre-training tasks were proposed, one based on multi-view contrastive learning and the other on pseudo-attribute classification. Another recent approach exploits the power of the CLIP model [41]; it is fine-tuned on more specific vision-and-language data for the fashion domain [55].

In the context of fashion-oriented image captioning, only a few works have been proposed [14,56]. In particular, Yang et al. [14] presented a novel dataset for the task and an attribute-augmented training strategy based on reinforcement learning to improve the final results. In contrast, this paper presents a novel architecture that can exploit additional retrieved information in the form of textual attributes or noun chunks to enrich predicted captions effectively.

## 3. Proposed Method

This section first introduces some important preliminaries on image captioning, then provides an overview of the proposed architecture, and finally details how it is augmented with a knowledge retrieval component and fully attentive gates.

### 3.1. Preliminaries

The majority of approaches for the image captioning task employ an autoregressive language model, which is conditioned on visual features extracted from the input images. The language model predicts the next word, given the previous words and the image features, i.e., it models a probability distribution

(1) $$p(w_{\tau }|w_{k<\tau },v),$$

where v is an input image, τ indicates time, and ${\left\{w_{\tau }\right\}}_{\tau }$ is the sequence of words comprising the generated caption.

Datasets for tackling the task in a fully-supervised manner are composed of image–caption pairs $D={\left\{\left(v_{i},t_{i}\right)\right\}}_{i}$, by means of which the language model is trained so as to generate sentences that should reproduce the ground-truth sequences. This is done by conditioning the model on ground-truth sub-sequences and training using a time-wise cross-entropy (XE) loss, as follows:

(2) $$L\left(\theta \right)=-E_{x∼D}\sum_{\tau }\log p\left(w_{\tau }|{\hat{w}}_{k<\tau },v,\theta \right),$$

where θ indicates the set of parameters of the model and $t={\left\{{\hat{w}}_{t}\right\}}_{t}$ is the ground-truth sequence. Optionally, a fine-tuning stage employing reinforcement learning with sentence-level rewards can be employed to increase the alignment of generated descriptions with a metric or to condition the behavior of the model with respect to global objectives [21,30].

### 3.2. Overview of Our Approach

The proposed approach augments the aforementioned schema by employing the external memory of textual data, from which relevant metadata about the input images can be retrieved and exploited at both generation and prediction times. Formally, the probability produced by the model is recast as

(3) $$p(w_{\tau }|w_{k<\tau },v,M),$$

where *M* represents the external memory, accessed through a retrieval component on the basis of the visual features v. The memory is built so to contain slots of textual information, each associated with a training image, so that textual pieces associated with similar images can be retrieved at the evaluation time. The retrieval part is based on a *k*-nearest-neighbor search between the input image (which acts as a query *q*) and all the images $x_{i}$ in the external memory *M*. The goal is to find the most similar images with respect to a score based on the inner product similarity between the embeddings of the images, i.e.,

(4) $$s\left(q,x_{i}\right)=\mathrm{Embed}{\left(q\right)}^{T}\mathrm{Embed}\left(x_{i}\right),$$

where Embed(·) is a visual encoder that transforms each image into a global feature vector. The images in the memory are sorted decreasingly by the similarity score s(·,·) and, finally, the best *k* images are retrieved to return the information related to them available in the external memory, such as their caption, category, and attributes. Noticeably, the presented approach offers better description performance without increasing the number of trainable parameters of the network, which inherently would imply heavier training costs.

**Figure 1:**
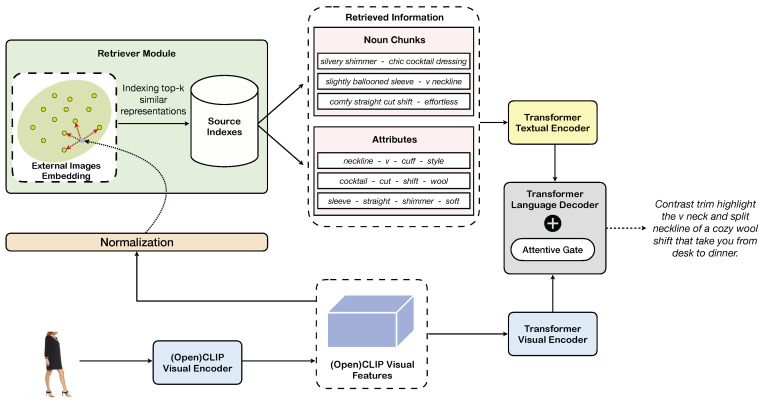
Overview of our approach for fashion-oriented image captioning.

From an architectural design point of view, the model introduced in this article is a fully attentive transformer [25] encoder–decoder network, which is adapted to support the inclusion of textual data retrieved from the memory. Specifically, the architecture comprises two encoders, one to process visual features and one to process textual items retrieved from the external memory. The input image is encoded using a vision transformer (ViT) [35] encoder trained à-la-CLIP [41]. The full grid of intermediate features coming from the last layer of the ViT is used as the input of the captioning encoder, while the feature vector of the [CLS] token, which provides global information, is employed as a query for the retrieval module, after $\ell_{2}$ normalization. The textual encoder, instead, is fed with textual items retrieved from the external memory. A transformer decoder, finally, is in charge of generating the output sentence by jointly attending the visual encoder output, which provides visual and contextual information, and that of the textual encoder, which provides knowledge retrieved from the memory. An overview of the proposed architecture is shown in Figure 1.

### 3.3. Knowledge Retrieval Architecture with Fully Attentive Gates

The input of the visual encoder consists of the flattened sequences of grid feature vectors coming from the CLIP encoder, which are linearly projected to match the internal dimensionalities of the transformer layers. The resulting vectors are then passed through a sequence of encoder layers, each of which performs self-attention and is followed by a feed-forward network. In the encoder, attention operations are not masked to allow bidirectional encoding of input image features with complete connectivity.

**Figure 2:**
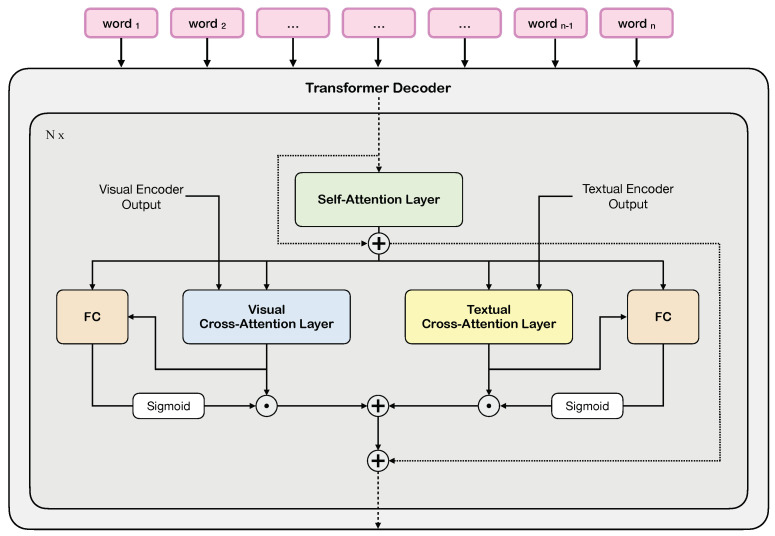
Architecture of the decoder, with attentive gates to integrate information coming from external memory.

The goal of the decoder is, instead, to predict a time-wise distribution over the vocabulary while being conditioned on both the input image and retrieved textual information. The input text from the ground-truth description, here, is tokenized using byte pair encoding(s) (BPE) [57] embedded into a vector space. The embedded vectors are then passed through a sequence of decoder layers, each of which performs a self-attention operation to attend to the input subsequence encoding the past context. In addition, it performs two cross-attention operations, respectively, with (*i*) the last layer of the textual encoder, which encodes retrieved textual information, and (*ii*) with the last layer of the visual encoder, which brings visual information about the input image (see Figure 2). Crucially, the same queries are employed for the cross-attention over the visual features and for the cross-attention over the encoded retrieved items from the memory. As in the classical transformer design, each decoder layer also contains a feed-forward network. In the decoder, a causal attention mask is employed to allow autoregressive operations and token embeddings of the last layer are used to predict the next token.

Given the input sequence of tokens, ${\left\{w_{i}\right\}}_{i}=\left\{w_{0},\ldots ,w_{i},\ldots ,w_{T}\right\}$, the set of retrieved textual information, i.e., ${\left\{z_{i}\right\}}_{i}=\left\{z_{0},\ldots ,z_{i},\ldots ,z_{N}\right\}$, as encoded by the textual encoder, and the set of visual features, i.e., ${\left\{v_{i}\right\}}_{i}=\left\{v_{0},\ldots ,v_{i},\ldots ,v_{M}\right\}$, as encoded by the visual encoder, the operations performed by the decoder can be written as follows:

(5) $$\begin{array}{cc} {\tilde{w}}_{t}^{l} & =\mathrm{MSA}(w_{t},{\left\{w_{i}\right\}}_{i=1}^{t}) \end{array}$$

(6) $$\begin{array}{cc} {\tilde{w}}_{t}^{m} & =\alpha_{t}\cdot \mathrm{MCA}\left({\tilde{w}}_{t}^{l},{\left\{z_{k}\right\}}_{k}\right)+\beta_{t}\cdot \mathrm{MCA}\left({\tilde{w}}_{t}^{l},{\left\{v_{k}\right\}}_{k}\right), \end{array}$$

where *k* indicates a generic item from the set of retrieved texts, *t* is the *t*-th element of the sequence of tokens, ${\left\{w_{i}\right\}}_{i=1}^{t}$ is the sequence of tokens up to the *t*-th element, MSA(x,y) indicates a multi-head self-attention with *x* mapped to query and *y* mapped to key values, and MCA(x,y) is the multi-head cross-attention with *x* as the query and *y* as the key values. The first equation refers to a self-attention between tokens of each retrieved caption, where the dependency on single tokens is omitted for readability. The last equation refers to the cross-attention operations with retrieved captions and textual features. Here, given ${\tilde{w}}_{t}^{l}$ as query, all tokens from all retrieved captions are attended.

The outputs of the two cross-attentions over the external memory and the visual features are combined using two learned sigmoidal and fully attentive gates α and β, which allow the model to choose between visual context and retrieved items. These are obtained by comparing the input and output of each of the cross-attention layers through a linear projection and using a sigmoidal activation to constraint their numerical range between 0 and 1. Formally,

(7) $$\begin{array}{c} \alpha_{t}=\sigma \left(\mathrm{FC}\left(\left[{\tilde{w}}_{t}^{l};\mathrm{MCA}\left({\tilde{w}}_{t}^{l},{\left\{z_{k}\right\}}_{k}\right)\right]\right)\right) \end{array}$$

(8) $$\begin{array}{c} \beta_{t}=\sigma \left(\mathrm{FC}\left(\left[{\tilde{w}}_{t}^{l};\mathrm{MCA}\left({\tilde{w}}_{t}^{l},{\left\{v_{k}\right\}}_{k}\right)\right]\right)\right), \end{array}$$

where FC indicates a fully connected layer with an output dimensionality of one, and $\left[\cdot ,\cdot \right]$ indicates tensor concatenation.

As it might be noticed, gradients are not backpropagated into the external memory, which is critical to the scalability of our technique. Following standard practice in image captioning, the model is trained with a time-wise cross-entropy loss over ground-truth captions (see Equation (2)).

## 4. Experimental Evaluation

In this section, quantitative and qualitative experiments are performed to validate the proposed solution with respect to carefully designed baselines and other state-of-the-art approaches. First, all implementation details, the dataset, and the metrics employed in the experimental evaluation are described.

### 4.1. Dataset

The model is trained and evaluated on the FAshion CAptioning Dataset (FACAD) [14], which is the biggest dataset available for fashion-oriented image captioning. As reported in [14], some other vision and language datasets are available in the domain of fashion, such as Fashion IQ [9], but all have a lower number of image–text pairs and have not been directly proposed for the captioning task. Specifically, FACAD contains 993k high-resolution images described by 130k fine-grained fashion-related captions, with 6∼7 images for each clothing item. Moreover, the dataset contains a list of 990 attributes and 78 categories to label each item with detailed information. The original splits provided by the authors of the dataset are used, with approximately 794k image–description pairs for training, 99k for validation, and the remaining 100k for testing. As textual retrievable items, either training captions or attributes annotated in the dataset are employed to populate the external memory.

### 4.2. Metrics

Following the captioning literature, the performance of the proposed model is evaluated in terms of standard captioning metrics, such as BLEU [58], METEOR [59], ROUGE [60], and CIDEr [61]. Captioning metrics are computed by employing the Microsoft COCO evaluation tool available at https://github.com/tylin/coco-caption, accessed on 15 January 2023. Additionally, following [14], a mean average precision (mAP) score computed over generated attributes is employed to evaluate how many times ground-truth attributes are named in the generated captions.

**BLEU.** It is an old classic measure for assessing the quality of the automatically generated text. This metric gauges how closely a machine’s output and a human’s output line up. A high-scoring description should be a perfect match for the length and wording of the ground-truth sentences. Its computation encompasses two different factors, i.e., a brevity penalty term that penalizes sentences that are shorter with respect to the reference ones, and a modified *n*-gram precision function, which is then employed to compute the final score.

**METEOR.** This measure was adopted to compensate for the shortcomings of the BLEU metric as it does not account for recall and only allows exact *n*-gram matching. It considers possible pairings of unigrams between the generated sentence and the reference one, even in absence of rigorous equality between them. The metric is based on the combination of precision and recall via a harmonic-mean, correlated with a penalty factor.

**ROUGE.** It is a formal *n*-gram recall metric that compares a candidate description against a group of reference summaries. It could be considered complementary to the BLEU score. It relies on the length of the *n*-gram, and the maximum number of *n*-grams co-occurring in the generated caption and the set of reference sentences.

**CIDEr.** This metric is based on term frequency–inverse document frequency (TF–IDF) computation and assigns a weight to each *n*-gram in a sentence depending on how frequently it appears in the corpus. It is formulated on the idea that the set of reference captions would commonly contain *n*-grams that are pertinent to the image. However, the inverse document frequency term assigns a lower weight to *n*-grams that are redundant throughout the entire dataset (i.e., in the reference captions of various images) since they are less likely to be instructive or important.

**mAP.** This metric is widely used in many deep learning tasks and it has a different meaning and computation depending on the application. In the context of fashion-oriented image captioning, the idea is to investigate how many attributes in the ground-truth captions are also in the generated descriptions, so the task is similar to multi-label classification. In this case, the mean average precision is the mean over all the attributes of the average precision of every single attribute. To guarantee a fair comparison with previous literature, the mAP metric is computed by using the source code provided by the authors of [14] and released at https://github.com/xuewyang/Fashion_Captioning, accessed on 15 January 2023.

### 4.3. Implementation Details

**Training Details.** To represent words of both the input sentences and the retrieved inputs, byte pair encoding (BPE) [57] is used with a vocabulary of 49,408 tokens. The word positions are represented by standard sinusoidal positional encodings [25]. The maximum length of the output token sequence is limited to 30 tokens to improve efficiency. To extract image features, different ViT-based visual encoders trained à-la-CLIP are employed. Specifically, image features are extracted from ViT-B/32 and ViT-L/14 visual models of both the standard CLIP architecture (https://github.com/openai/CLIP, accessed on 15 January 2023) [41] and the open-source implementation of CLIP (i.e., OpenCLIP (https://github.com/mlfoundations/open_clip, accessed on 15 January 2023) [62]), which has been trained with a post-ensemble method for improving robustness to out-of-distribution samples. The OpenCLIP architectures employed in the experiments were trained on the LAION-2B composed of 2 billion image–text pairs obtained by filtering the English pairs of the LAION-5B dataset [63].

Both visual features and textual tokens are projected into the input dimensionality of the model, which is set to d=384. The presented captioning model has 3 layers in both the encoder and decoder, with 6 attention heads and a feed-forward layer size of 1536. The external memory encoder has the same number of heads and dimensionalities as the rest of the model, except for a single transformer layer. Training is performed by using Adam [64] as the optimizer. Specifically, the model is trained up to 150,000 iterations using a cross-entropy loss and a batch size of 64, following the typical transformer learning rate scheduling strategy [25] with a warm-up equal to 6000 iterations and a learning rate multiplier of 0.5. During caption decoding, the beam search algorithm is used with a beam size equal to 5 in all of the experiments. After training, the best model is selected based on the highest CIDEr score achieved on the FACAD validation set.

**Retrieval Index.** The retrieval index is built using the whole FACAD training set. To avoid the risk of overfitting, during the training phase, the current view’s image is not considered when retrieving information from the external memory. The approximated *k*-nearest neighbors (*k*NN) search is used instead of an exact *k*NN search to reduce the computational load of our approach. In particular, the Faiss library (https://github.com/facebookresearch/faiss, accessed on 15 January 2023) [65] is employed with a graph-based hierarchical navigable small world (HNSW) index with 32 links per vertex, which has a size of almost 3 GB when containing the image features obtained using the ViT-L/14 version of the OpenCLIP vision encoder. Because of the small-scale size of the dataset, no transformation or quantization is applied to the computed index, which could be useful to reduce its size and scale to bigger datasets. In the experiments, the *k*NN search is run on the CPU thanks to the optimization provided by the Faiss library. The number of neighbors to be retrieved in *k*NN queries is set to 10 in each experiment.

### 4.4. Model Ablation and Analysis

It is firstly convenient to investigate which textual information should be stored in the external memory. In Table 1, two options are compared: (*i*) using the attributes obtained from the original metadata of the dataset, and (*ii*) using the noun chunks extracted from each training caption, employing the spaCy library (https://spacy.io/, accessed on 15 January 2023) for natural language processing. In this analysis, both CLIP and OpenCLIP ViT-L/14 models are employed. As can be seen, the noun chunks strategy overcomes the attributes strategy with CLIP and OpenCLIP visual features, with an improvement of almost 4 CIDEr points in both settings of the FACAD test set. This is a positive experimental finding because it relieves the need to manually annotate metadata and proves that information extracted from the captions can be proper content for the external memory. Throughout the rest of the experimental analysis, the retrieval strategy based on noun chunks is employed.

In Table 2, the effectiveness of the proposed retrieval-augmented architecture is validated using different visual features. In addition to the ViT-L/14 models, experimental results are also reported using the ViT-B/32 version in both CLIP and OpenCLIP settings. To validate the benefits of using an external knowledge-based memory from which to retrieve additional information during the generation process, a standard encoder–decoder transformer model is trained with the same hyperparameters and settings used to train the complete architecture. This model can be considered as an ablation of the proposed retrieval-augmented model without the retrieval component. In the baseline setting, both the OpenCLIP models overcome the respective CLIP ones, obtaining an improvement of 8.4 CIDEr points with the ViT-B/32 visual encoder and 13.7 CIDEr points with the ViT-L/14 one. Surprisingly, the OpenCLIP ViT-B/32 vision encoder surpasses the CLIP ViT-L/14 model by a good margin. All retrieval-based architectures show huge improvements with respect to the baseline models—i.e., up to 32.3 CIDEr points in the OpenCLIP ViT-B/32 setting. Overall, the best results are achieved by the model trained with visual features extracted from OpenCLIP ViT-L/14 with 84.5 CIDEr points on the FACAD test set.

### 4.5. Comparison to the State-of-the-Art

Having identified the best model configuration (i.e., the one trained with OpenCLIP ViT-L/14 visual features and noun chunks retrieval strategy), Table 3 compares the proposed approach with other state-of-the-art models trained on the FACAD dataset. In particular, the experimental comparison includes (*i*) models trained with cross-entropy loss, (*ii*) models trained with reinforcement learning, and (*iii*) transformer-based models trained with the same visual features of the proposed approach. For the first category, the comparison includes show, attend, and tell [12], up–down [13], and LBPF (“Look Back and Predict Forward”) [22], which employ recurrent neural networks and attention over CNN features or region-based features extracted from object detectors. It further includes ORT (“Object Relation Transformer”) [29], which is based on fully attentive mechanisms and SRFC (“Semantic Rewards guided Fashion Captioning”) [14] in its baseline configuration without rewards. For the second category, it considers the self-critical sequence training approach (SCST) [21], which uses the REINFORCE algorithm to directly optimize the CIDEr metric and SRFC [14] in its final version, which employs attribute-level semantic and sentence-level semantic rewards to improve the captioning quality. Finally, the experimental analysis also includes the results of a standard transformer-based model and two additional state-of-the-art competitors based on the transformer architecture, namely the meshed-memory transformer ($M^{2}$ transformer) [30] and CaMEL (“Captioner with Mean tEacher Learning”) [33]. The latter is trained with the same visual features used by the solution introduced in this article with cross-entropy loss only (i.e., OpenCLIP ViT-L/14).

As can be seen, the proposed approach outperforms all of the compared methods according to BLEU, CIDEr, and mAP metrics, reaching 84.5 CIDEr points on the FACAD test split. This corresponds to an improvement of about 30 CIDEr points over the standard transformer model and other transformer-based competitors, thus confirming the effectiveness of augmenting a captioning architecture with external knowledge retrieval. From the analysis, it is worth noting that the proposed architecture is trained with cross-entropy loss only, differently, for example, from the SRFC model [14], which is further optimized with a reinforcement learning strategy. Nevertheless, the presented model is able to outperform the SRFC architecture by more than 40 CIDEr points, while being slightly worse in terms of METEOR and ROUGE metrics.

### 4.6. Computational Analysis

To evaluate the computational cost of the proposed architecture, it is beneficial to analyze the training requirements of the baseline transformer model without any external retrieval component or additional textual encoder. This requires around 120 h using two NVIDIA GeForce RTX 2080 Ti GPUs and ViT-L/14 features. When adding the external memory, the retrieval component, and the additional textual encoder, the training time increases by around 16 h, which roughly corresponds to a 14% increase. It should be noted that the time required to retrieve and extract textual data from the external memory also depends on the number *k* of neighbors to be retrieved. In all experiments, *k* is set to 10, leaving the analysis of the performance-efficiency ratio of different *k* values for future works.

### 4.7. Qualitative Results

**Figure 3:**
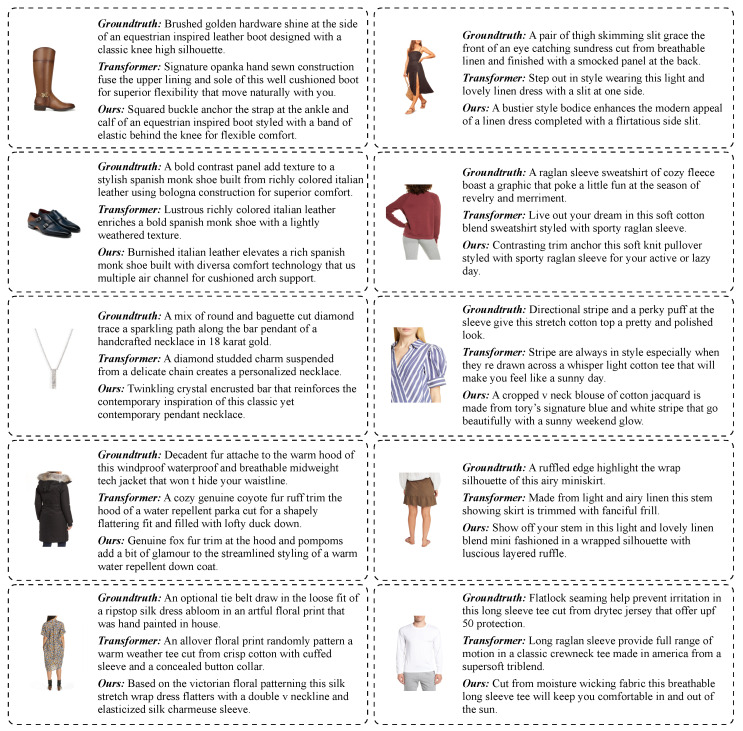
Qualitative results of the proposed approach with and without the retrieval strategy, compared with ground-truth sentences.

In Figure 3, some qualitative results are reported, comparing captions generated by the proposed approach with those generated by the transformer-based baseline, which does not employ external memory. For completeness, the ground-truth description of each of the images is also included. Thanks to the retrieved textual information, the captions generated by the presented model are more fine-grained than those provided by the baseline, allowing the proposed captioner to describe each item with precise details, such as the kind of fabric, the presence of specific patterns, and the type of outfit.

**Figure 4:**
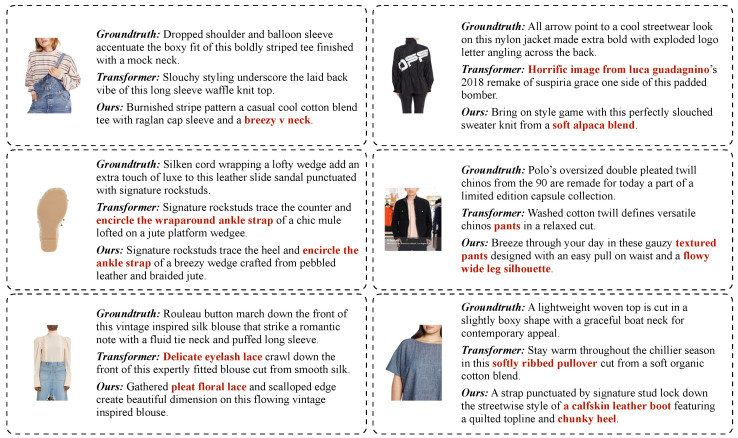
Failure cases of the presented solution, both in the baseline and retrieval settings.

While the retrieval component can be helpful during the generation, sometimes it may fail and return textual information that does not match the visual content of the image, thus affecting the quality of the generation. This is qualitatively shown in Figure 4. As can be seen, failures might occur in the presence of single objects placed in difficult poses, to garble the network in guessing the right categories they belong to. This situation is visually represented in the image with a shoe sole, which deceives the network to predict a human ankle. Wrong details might also be generated because retrieved items have similar but slightly different characteristics (e.g., colors, blends, or textures). Moreover, the background or the presence of other objects in the image may impact the quality of the generated caption, such as in the image in the right part of the second row. The proposed model describes only the pants but the main item in the image is the shirt.

## 5. Conclusions

This work investigated the use of a retrieval component applied to the fashion captioning task. In particular, it presented a transformer-based network that employs *k*NN-augmented attention layers to help the model during sentence generation, using textual information retrieved from external memory. From an architectural point of view, the proposal consists of an encoder–decoder transformer-based network augmented with an additional textual encoder to process items retrieved from the external memory. Then, the decoder attends to the outputs of both encoders through cross-attention operations. The contributions of the visual and textual encoders are balanced through sigmoidal fully attentive gates. Experimentally, the proposed approach surpassed the state-of-the-art on the largest dataset for the available fashion captioning, FACAD, demonstrating the appropriateness of the model and its capacity to retrieve significant data and, thus, generate fine-grained descriptions of fashion articles.

Overall, this work demonstrates the benefits of employing external memories when applying image captioning to a specialized domain, such as the fashion one. Future works in this direction will include design variations of the fully attentive gates, the exploration of other fine-grained domains, and the extension of the approach to large-scale datasets for generic image captioning.

## Figures and Tables

**Table 1:** Ablation study results on the FACAD validation and test splits using different retrieval strategies.

		Validation Set					Test Set				
	Retrieval	B-1	B-4	M	R	C	B-1	B-4	M	R	C
**CLIP ViT-L/14**	attributes	**26.2**	10.0	**10.9**	**21.4**	75.6	24.8	8.1	**10.2**	20.2	63.1
	chunks	26.1	**10.4**	10.8	21.3	**78.9**	**25.0**	**8.3**	**10.2**	**20.3**	**67.0**
**OpenCLIP ViT-L/14**	attributes	28.6	12.1	12.3	23.4	93.1	27.4	10.2	**11.6**	**22.4**	80.9
	chunks	**28.9**	**13.0**	**12.4**	**23.7**	**100.9**	**27.3**	**10.6**	11.5	22.3	**84.5**

**Table 2:** Ablation study results on the FACAD validation and test splits using different visual encoders.

		Validation Set					Test Set				
	Retrieval	B-1	B-4	M	R	C	B-1	B-4	M	R	C
**CLIP ViT-B/32**	**✗**	22.6	5.5	8.9	17.6	39.7	21.6	4.1	8.4	16.9	32.4
	**✓**	**24.5**	**9.2**	**9.9**	**19.9**	**69.3**	**23.5**	**7.5**	**9.3**	**18.9**	**57.3**
		(+1.9)	(+3.7)	(+1.0)	(+2.3)	(+29.6)	(+1.9)	(+3.4)	(+0.9)	(+2.0)	(+24.9)
**OpenCLIP ViT-B/32**	**✓**	24.5	7.4	10.0	19.9	55.2	23.2	5.2	9.3	18.2	40.8
	**✓**	**27.1**	**11.2**	**11.4**	**22.0**	**85.8**	**25.8**	**9.3**	**10.7**	**21.0**	**73.1**
		(+2.6)	(+3.8)	(+1.4)	(+2.1)	(+30.6)	(+2.6)	(+4.1)	(+1.4)	(+2.8)	(+32.3)
**CLIP ViT-L/14**	**✗**	24.1	7.1	9.8	19.2	52.3	22.7	5.0	9.0	18.0	39.3
	**✓**	**26.1**	**10.4**	**10.8**	**21.3**	**78.9**	**25.0**	**8.3**	**10.2**	**20.3**	**67.0**
		(+2.0)	(+3.3)	(+1.0)	(+2.1)	(+26.6)	(+2.3)	(+3.3)	(+1.2)	(+2.3)	(+27.7)
**OpenCLIP ViT-L/14**	**✗**	25.9	8.9	10.8	20.9	67.4	24.5	6.8	10.1	19.7	53.0
	**✓**	**28.9**	**13.0**	**12.4**	**23.7**	**100.9**	**27.3**	**10.6**	**11.5**	**22.3**	**84.5**
		(+3.0)	(+4.1)	(+1.6)	(+2.8)	(+33.5)	(+2.8)	(+3.8)	(+1.4)	(+2.6)	(+31.5)

**Table 3:** Comparison with the state-of-the-art on the FACAD test split. The † marker indicates models trained with the same visual features of the proposed approach (i.e., OpenCLIP ViT-L/14).

	B-1	B-4	M	R	C	mAP
Show, Attend, and Tell [12]	-	4.3	9.5	19.1	35.2	0.056
Up–Down [13]	-	4.4	9.7	19.6	36.9	0.058
LBPF [22]	-	4.5	9.5	19.1	36.4	0.055
ORT [29]	-	4.2	10.2	19.9	36.7	0.061
SRFC [14]	-	4.4	9.8	20.2	35.6	0.058
SCST [21]	-	5.6	11.8	22.0	39.7	0.080
SRFC (RL-fine-tuned) [14]	-	6.8	**13.2**	**24.2**	42.1	0.095
Transformer ${}^{†}$	24.5	6.8	10.1	19.7	53.0	0.238
$M^{2}$ Transformer ${}^{†}$ [30]	24.7	6.8	10.4	19.9	53.3	0.237
CaMEL ${}^{†}$ [33]	25.0	7.0	10.7	20.4	55.0	0.241
**Ours**	**27.3**	**10.6**	11.5	22.3	**84.5**	**0.248**

## Data Availability

The Fashion Captioning dataset (FACAD) is publicly available at https://github.com/xuewyang/Fashion_Captioning, accessed on 15 January 2023.
